# Three *Klebsiella pneumoniae* lineages causing bloodstream infections variably dominated within a Greek hospital over a 15 year period

**DOI:** 10.1099/mgen.0.001082

**Published:** 2023-08-29

**Authors:** Ayorinde O. Afolayan, Anastasia Rigatou, Hajo Grundmann, Angeliki Pantazatou, George Daikos, Sandra Reuter

**Affiliations:** ^1^​ Institute for Infection Prevention and Control, Medical Center - University of Freiburg, Freiburg, Germany; ^2^​ Department of Microbiology, Laiko General Hospital, Athens, Greece; ^3^​ First Department of Medicine, Laiko General Hospital, National and Kapodistrian University of Athens, Athens, Greece

**Keywords:** carbapenem resistance, colistin, Greece, *Klebsiella pneumoniae*, local surveillance

## Abstract

Carbapenem-resistant *Klebsiella pneumoniae* (CRKP) has emerged as a major clinical and public health threat. The rapid dissemination of this pathogen is driven by several successful clones worldwide. We aimed to investigate the CRKP clonal lineages, their antibiotic resistance determinants and their potential transmissions in a tertiary care hospital located in Athens, Greece. Between 2003 and 2018, 392 CRKP isolates from bloodstream infections were recovered from hospitalized patients. Whole genome sequencing (WGS) was performed on the Illumina platform to characterize 209 of these isolates. In total, 74 % (*n*=155) of 209 isolates belonged to three major clonal lineages: ST258 (*n*=108), ST147 (*n*=29) and ST11 (*n*=18). Acquired carbapenemase genes were the mechanisms of resistance in 205 isolates (*bla*_KPC_, *n*=123; *bla*_VIM_, *n*=56; *bla*_NDM_, *n*=20; *bla*_OXA-48_, *n*=6). Strong associations (*P*=0.0004) were observed between carbapenemase genes and clonal lineages. We first isolated *bla*_VIM-1_-carrying ST147 strains during the early sampling period in 2003, followed by the emergence of *bla*_KPC-2_-carrying ST258 in 2006 and *bla*_NDM-1_-carrying ST11 in 2013. Analysis of genetic distances between the isolates revealed six potential transmission events. When contextualizing the current collection with published data, ST147 reflected the global diversity, ST258 clustered with isolates representing the first introduction into Europe and ST11 formed a distinct geographically restricted lineage indicative of local spread. This study demonstrates the changing profile of bloodstream CRKP in a tertiary care hospital over a 15 year period and underlines the need for continued genomic surveys to develop strategies to contain further dissemination. This article contains data hosted by Microreact.

## Data Summary

The raw sequence data and assemblies have been deposited in the European Nucleotide Archive under the project ID PRJEB58216 (Table S1, available in the online version of this article). A collection has been created in Pathogenwatch (https://pathogen.watch/collection/qqmku6evab72-cpkp-athens-greece) to explore the data. Collections were also created with global isolates for ST147 (https://pathogen.watch/collection/pll287yfjynm-daikos-greece-st147-and-global-collection), ST258 (https://pathogen.watch/collection/l8paxhiqfa1f-daikos-greece-st258-and-global-collection) and ST11 (https://pathogen.watch/collection/ezyujarj6aw5-daikos-greece-st11-and-global-collection).

**Impact Statement**

Carbapenem-resistant *Klebsiella pneumoniae* (CRKP) infections are a significant clinical and public-health threat worldwide. We recovered 392 CRKP bloodstream isolates from patients admitted to a tertiary referral hospital in Athens, Greece, between 2003 and 2018 and analysed 209 of those by whole genome sequencing. Three high-risk lineages were predominantly circulating within this hospital – ST147, ST258 and ST11 – which emerged sequentially in 2003, 2006 and 2013, and carried mainly the *bla*_VIM_, *bla*_KPC_ and *bla*_NDM_ carbapenemase genes respectively. More than 30 % of CRKP blood isolates co-carried extended-spectrum beta-lactamase genes, aminoglycoside-modifying enzyme genes and mutations conferring resistance to colistin, one of the last treatment options for CRKP infections. These high-risk clones were well established in the hospital and have persisted over time. Based on whole genome analysis and SNP distances, several intrahospital putative transmission events were detected. Our findings have important clinical and infection control implications. Understanding the local molecular epidemiology of CRKP is essential in designing interventions to curtail its spread. If a high-risk clone is promptly identified and its initial spread in a given hospital is interrupted by a ‘search and destroy’ strategy, endemicity can be prevented. Should similar CRKP clones circulate between hospitals in the same country, a rigorous national intervention programme may be more efficient to contain CRKP by minimizing re-introduction into a hospital via transferred patients. Also, active surveillance of carbapenemase genes and other key antimicrobial resistance genes will direct physicians in making treatment decisions and in implementing a clone-specific antibiotic stewardship programme, especially with respect to ceftazidime/avibactam use, which is not active against *bla*_VIM_ and *bla*_NDM_ producers.

## Introduction

Carbapenem-resistant *Klebsiella pneumoniae* (CRKP), recognized by WHO as being among the pathogens of critical priority [1], has been established in many hospitals worldwide causing serious infections associated with increased morbidity and mortality [2–4]. Over the last decade, the incidence and prevalence of CRKP as well as other carbapenem-resistant *Enterobacterales* have been increasing at an alarming rate in Europe and other parts of the world [5]. Carbapenem resistance is mainly mediated by carbapenemase production encoded by genes carried on mobile genetic elements [2, 3]. The most common carbapenemase genes are those coding for *K *. *pneumoniae* carbapenemase (*bla*_KPC_), New Delhi metallo-beta-lactamase (MBL) (*bla*_NDM_), Verona integron-encoded MBL (*bla*_VIM_) and oxacillinase-48-like carbapenemases (*bla*_OXA-48-like_) [2, 3]. The genetic characteristics of CRKP populations (e.g. clones, capsule, mobile elements, and antimicrobial resistance and virulence genes) have been changing over time with substantial geographical variation [6, 7].

In Europe, CRKP has become endemic in many countries [8, 9]. In Greece, the emergence of *K. pneumoniae* producing carbapenemase was first reported in 2003 [10]. Since then, the epidemiology has been changing with sequential emergence and spread of different clones producing VIM, KPC, NDM and OXA-48-like carbapenemases [11, 12]. At present, a polyclonal epidemic of CRKP is affecting major hospitals across the country and according to 2022 ECDC-WHO Antimicrobial Resistance Surveillance report, the proportion of invasive CRKP isolates in Greece is 66 %, the highest among European countries [13].

Several studies have examined the molecular epidemiology of CRKP in Greece in the past [14–18]. These reports, however, covered limited time periods, have relied on less discriminating typing methods and none have used whole genome sequencing (WGS). In the present study, we performed WGS in 209 CRKP blood isolates derived from a tertiary care hospital over a 15 year period in order to obtain a snapshot of clonal evolution of CRKP strains, determine the antimicrobial resistance mechanisms and examine their potential nosocomial spread.

## Methodology

### Study setting and patient population

This is a retrospective study conducted between 2003 and 2018 in Laiko General Hospital in Athens, Greece. Laiko is a tertiary-care, 500 bed hospital with 50 000 admissions per year serving a population of 2 million of the Athens Metropolitan area. The hospital includes the following wards: three general medicine, two general surgery, one vascular surgery, one orthopaedic, one urology, one gynaecology, one ENT/ophthalmology, one haematology, one nephrology, one cardiology, one transplant, one intensive care unit (ICU) and one coronary care unit (CCU). The majority of the rooms are three- and two-bed. Anonymized clinical data and epidemiological data (including dates of hospital admission) were retrieved from hospital records. Pertinent information including date of positive culture, patient age, gender and hospital ward location of the patient at the time of infection were recorded. CRKP bloodstream infections have been occurring during the study period with incidence ranging from 0.04 to 0.35 per 1000 patient-days. The study protocol was approved by the Institutional Review Board with a waiver of informed consent (17/11/2017; ref. 1479).

### Antibiotic susceptibility testing and phenotypic determination of carbapenem resistance

All consecutive *K. pneumoniae* isolates recovered from patients with bloodstream infection (the first isolate per patient) with meropenem minimum inhibitory concentration (MIC) ≥0.5 mg l^−1^ and/or imipenem disc diameter of ≤23 mm were identified from the Laboratory Informational System and were retrieved from deep refrigeration for further studies. Species identification and antimicrobial susceptibility testing (AST) were performed using a MicroScan autoSCAN-4 system (Beckman Coulter). Following species identification, isolates had been stored in skimmed milk at −70 °C. Susceptibility to meropenem, tigecycline, colistin and ceftazidime/avibactam was re-evaluated by a broth microdilution method (Thermo Scientific Sensititre) using *Escherichia coli* ATCC 25922 and *E. coli* ATCC 13846 as control strains. Susceptibility to other antibiotics besides meropenem, tigecycline, colistin and ceftazidime/avibactam was tested using the MicroScan autoSCAN-4 system (Beckman Coulter). European Committee of Antimicrobial Susceptibility Testing (EUCAST) breakpoints were used for interpretation of the results (http://www.eucast.org/clinical_breakpoints). The isolates were defined as carbapenem resistant using the Centers for Disease Control (CDC) definition (isolates that phenotypically test resistant to any carbapenem or harbour a carbapenemase encoding gene or test positive for carbapenemase production) [19].

### Molecular determination of carbapenem resistance genes

Genomic DNA for PCR was prepared with a QIAamp DNA Mini Kit (Qiagen). The presence of carbapenemase encoding genes (*bla*_KPC_, *bla*_VIM_, *bla*_NDM_ and *bla*_OXA-48_) was examined by PCR using specific primers as previously described [10, 18, 20, 21]. Only if none of the mentioned carbapenemase genes were detected, screens for other carbapenemase genes such as *bla*_IMP_ or *bla*_OXA-48-like_ wre carried out. We used the fully characterized strains HPIVIM, HPIKPC, HPINDM and HPIOXA-48 obtained from the Hellenic Pasteur Institute as positive controls.

### DNA extraction, library preparation and WGS

From a total of 392 CRKP recovered during the study period, 209 representative isolates were selected for WGS (Tables S1 and S2). Since the study did not receive any funding, representative isolates were chosen for sequencing. Following the initial detection of carbapenemase producers, all isolates of the first 2 years were included. Afterwards, the first and last isolate per month were selected, if such isolates were available, and when more than two were available, a third isolate from the same month, the closest in time of isolation with the first or the last one, was also included in order to detect any clonal spread. Genomic DNA was extracted following a protocol described previously (https://lifescience.roche.com/documents/High-Pure-PCR-Template-Preparation-Kit.pdf), using the High Pure PCR Template Preparation Kit (Roche). DNA libraries were prepared using a Nextera DNA Flex Library Preparation Kit (Illumina). Paired-end sequencing (300 cycle v2, 2×150 reads) was performed on an Illumina MiSeq platform (Illumina).

### Genomic analyses

### Sequencing quality control, mapping and assembly

Raw sequence reads were checked for the corresponding species using the Kraken v0.10.5-beta [22] and the minikraken 4 GB reference database. Isolates were mapped to MGH78578, as well as ST147 to JACTAR01, ST258 to CP006923.1, and ST11 isolates were mapped to NZ_CP021685.1 using smalt version 0.7.6 (https://www.sanger.ac.uk/science/tools/smalt-0) [23]. MGH78578 was chosen as a reference because it has the most complete annotation of the chromosome and accompanying plasmids universally known for *K. pneumoniae* [24].

JACTAR01 (also known as HKP0064 with RefSeq accession GCF_014904025.1) is a completely annotated, extensively drug-resistant ST147 strain recovered from Germany [25]. CP006923.1 (RefSeq accession GCF_000598005.1) is a well-annotated carbapenem-resistant ST258 clinical reference strain previously used in the investigation of *bla*_KPC_-carrying *K. pneumoniae* ST258 evolution [26]. NZ_CP021685.1 is the closest reference in NCBI Refseq to all ST11 strains in this study, using the Bactinspector (https://gitlab.com/antunderwood/bactinspector) *closest_match* sub-command. Sequence reads were assembled using SPAdes v3.11.1 (http://cab.spbu.ru/software/spades/) [27] with kmer sizes of 21, 33, 55, 77, 99, 109 and 123. Assemblies were then filtered to only include contigs with a minimum of 500 bp. Multi-locus sequence typing (MLST) was carried out with mlst v2.10 (https://github.com/tseemann/mlst) using the respective typing schemes where applicable, and new sequence types (STs) were assigned. QC criteria included: allocation of reads to the respective expected genus, coverage greater than 30×, appropriate genome size (4–6 Mb), number of contigs fewer than 500, largest contig greater than 100 000 bp, N50 greater than 100 000 bp, and identification of MLST alleles. Average coverage was 50×, and average length of assemblies was 5.6 Mb, in 92 contigs, with N50 264 kb. Statistics and metadata for individual isolates can be found in Table S1.

### Phylogenetic tree reconstruction

For phylogenetic reconstructions, SNPs were filtered from the mapping data with GATK (https://gatk.broadinstitute.org/hc/en-us) [28], and only SNPs with at least four reads coverage and present in >75 % of reads were included. These variant filtered files were converted to a fasta file, where SNP sites and absent sites (N) were replaced compared to the reference genome. All isolates of a species were then combined to an alignment, and regions resembling mobile genetic elements were removed (https://github.com/andrewjpage/remove_blocks_from_aln). Recombination was removed using Gubbins [29]. SNP sites were extracted, and the resulting alignment was used to reconstruct a maximum-likelihood phylogeny with RAxML v8.2.4 [30].

### Serotyping, and the detection of antimicrobial resistance, virulence and plasmid replicon genes

The Kleborate pipeline (v2.0.4) (https://github.com/katholt/Kleborate) [31, 32] was used to screen genome assemblies for multilocus sequence (MLST), speciation, the prediction of capsular (K) and lipopolysaccharide (O antigen) serotype, virulence determinants, and antimicrobial resistance (AMR) determinants. Plasmid replicons were predicted using Abricate (https://github.com/tseemann/abricate) and the PlasmidFinder database [33]. Coverage and nucleotide identity cutoff of 100 and 100 %, respectively, were used for determining the existence of AMR genes, virulence genes and plasmid replicons.

### Genome annotation and assessment of the genetic environment of carbapenemase genes

Contigs carrying *bla*_KPC_, *bla*_VIM_ and *bla*_NDM_ carbapenem resistance genes, as well as contigs carrying *bla*_VEB-1_ and *mgrB* truncations were extracted from genome assemblies (using https://github.com/raymondkiu/bioinformatics-tools/blob/master/extract-contig.pl) and annotated using Bakta v1.0.4 [34]. TETyper (v1.1) [35] was used to identify structural variants of the Tn*3*-based transposon, Tn*4401* in *bla*_KPC_-carrying isolates. NDM-carrying genomes were screened against the ISfinder database (https://isfinder.biotoul.fr/) [36] for the insertion sequence IS*Aba125*.

### Statistics and visualization

Associations between carbapenemase genes and clonal lineages were calculated in R (v4.1.0) [37], using Fisher’s Exact Test for count data. MICs were converted to RIS (Resistant, Intermediate, Susceptible) according to EUCAST guidelines using the AMR R package [38]. Agreement between phenotypic and genotypic resistance for colistin, tigecycline, ceftazidime-avibactam and carbapenem was determined by calculating metrices such as concordance, true positives, true negatives, false positives and false negatives, using an R script (https://gitlab.com/-/snippets/2050300). Bar plots and other plots were generated in R (v4.1.0) [37], using the Tidyverse suite of packages [39], Plotme (v0.1.0; for Sunburst plots) [40], ggsankey (v0.0.9; for Sankey plots) [41] and Patchwork (v1.1.1; for plot combination) [42] packages. Annotated contigs were compared and visualized using Clinker (v0.0.21) [43]. Pathogenwatch (https://pathogen.watch) [44] was used to determine the global context of genomes derived from this retrospective Greek collection. Microreact [45] was also used to interactively visualize the placement of ST147 (https://microreact.org/project/sBQBwnNFwemsJS3GkgdUMk-st147-daikos-global-context), ST258 (https://microreact.org/project/cbq9gSPtNV89yeXf1igjUZ-st258-daikos-global-context) and ST11 (https://microreact.org/project/g7ykirHAVyFxETufRPQJnh-st11-daikos-global-context) genomes within a global context. The Interactive Tree of Life (ITOL) [46] was used to visualize the phylogenetic tree and metadata.

## Results

### Patient population and AMR profile

Of 209 selected cases, 127 (60.8 %) were male and 82 (39.2 %) were female. The mean patient age was 50 years (median, 49 years; range 31–78 years). Ninety-seven (46.4 %) infections occurred in internal medicine wards, 57 (27.3 %) in the ICU, 21 (10.0 %) in surgical wards, 15 (7.2 %) in the haematology unit, and the remaining 19 (9.1 %) in other wards. The median duration of hospitalization before the onset of bacteraemia was 16 days (interquartile range 8–32 days). In the majority of patients, the bacteraemia was secondary: 55 (26.3 %) patients had urinary tract infection, 38 (18.2 %) intravascular catheter-associated infection, 25 (12.0 %) hospital-acquired or ventilator-associated pneumonia, 19 (9.1 %) intra-abdominal infection, and 15 (7.6 %) skin and soft tissue infection. In 56 (26.8 %) cases, no definitive portal of entry could be detected (Table S1).

All isolates were resistant to penicillin-inhibitor combinations and extended-spectrum cephalosporins. The majority of the isolates were also resistant to ciprofloxacin (95.2 %), co-trimoxazole (89 %), amikacin (79.9 %), meropenem (78 %) and tigecycline (65.5 %). The most active drugs were gentamicin and colistin, although considerable numbers of isolates exhibited resistance to these agents: 37.8, and 34.9 % respectively (Table S1). Of note, after intensified colistin and tigecycline use, we observed a significant increase in resistance from 21.2 and 44.2 % in the period 2003–2010; 39.5 % (*P*=0.02) and 72.0 % (*P*<0.001) in the period 2011–2018 for colistin and tigecycline, respectively. Ceftazidime/avibactam did not have any activity against VIM and NDM isolates while all KPC and OXA-48 isolates were susceptible to this agent except for one KPC-23 isolate that exhibited resistance.

### Epidemiology and dynamics of CRKP in a Greek hospital

**Fig. 1.**
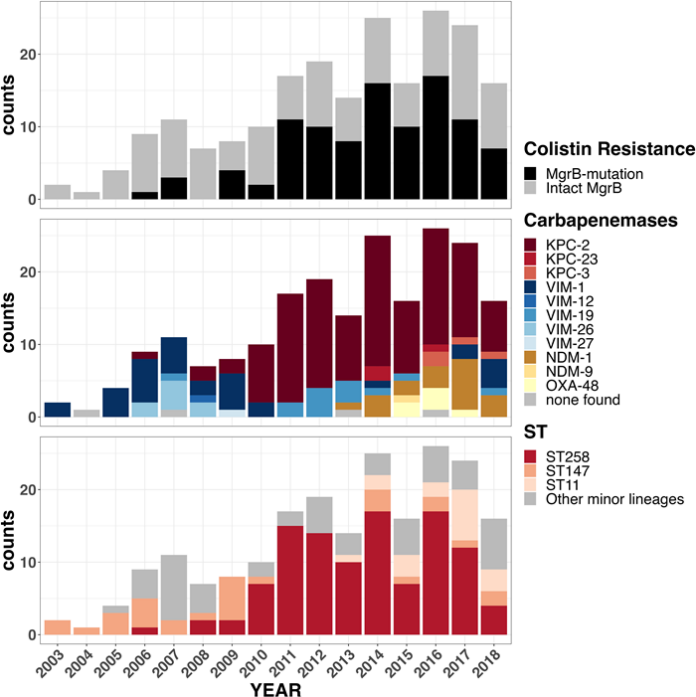
The distribution of CPKP lineages, carbapenemases and colistin mutated gene carried by strains within these lineages over the 15 year sampling period.

**Fig. 2.**
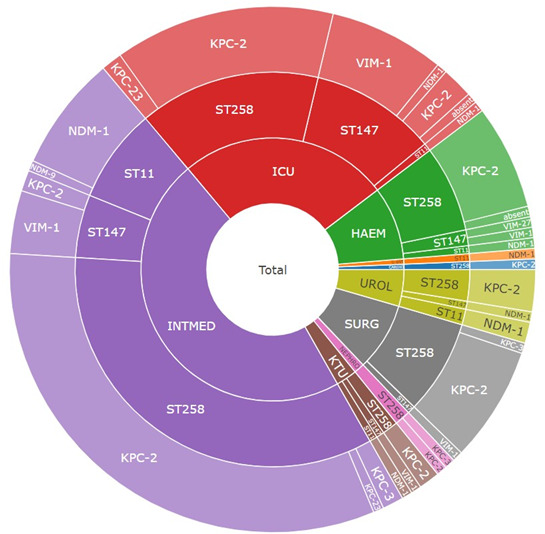
Sunburst diagram of the distribution of carbapenemase genes in isolates belonging to ST258, ST147 and ST11 recovered from patients admitted to nine wards. INTMED: Internal Medicine (1, 2, 3); KTU: Kidney Transplant Unit; NEPHRO: Nephrology; SURG: Surgical Wards (1, 2, 3); UROL: Urology; CARDIO: Cardiology; G-Ent: Gastroenterology; HAEM: Haematology; ICU: Intensive Care Unit.

Genomic analyses of 209 CRKP bloodstream strains (phylogroup Kp1) showed that 155 (74 %) belonged to the ST11 (*n*=18), ST147 (*n*=29) and ST258 (*n*=108) in varying frequencies over the sampling period (2003–2018) (Fig. 1). A strong association between lineages and carriage of carbapenemase genes were observed (*P*=0.0004) as a large proportion of ST147, ST258 and ST11 CRKP strains carried variants of the *bla*_VIM_, *bla*_KPC_ and *bla*_NDM_ carbapenemase genes, respectively (Table 1, Figs 2 and S1) Lineages were not restricted to particular wards (Fig. 2) but were found throughout the hospital. As had been detected by PCR, sequencing also showed a rise of ST147-VIM in frequency at the start of the sampling period (2003; Table S2). This lineage was then displaced by strains corresponding to an expanding ST258-KPC lineage. With the first *bla*_NDM_ detected in 2013, ST11 then emerged a decade after the start of sampling, and ST147 re-emerged in 2014, albeit at a lower frequency than that at the start of sampling, and with a change in carbapenemases, as *bla*_NDM_- and *bla*_KPC-2_-positive isolates were found as well. Isolates outside the main dominant lineages (including ST383, ST36, ST101, ST17, ST323, ST1237, ST307, ST39) carried a *bla*_VIM_ variant (*n*=35/54), *bla*_KPC-2_ (*n*=11) or *bla*_OXA-48_ (*n*=6) (Table S1).

**Table 1.** Overview of genomic characteristics of major lineages of CRKP circulating within a local tertiary hospital in Athens, Greece; the numbers in parentheses indicate the number of isolates

Sequence type	Carbapenemases (*n*)	ESBL (*n*)	Colistin	Yersiniabactin	T*n4401*
ST147 (*n*=29)	*bla*_VIM-1_ (20) *bla*_KPC-2_ (5) *bla*_NDM-1_ (2) *bla*_VIM-27_ (1)	*bla*_CTX-M-15_ (3) *bla*_CTX-M-3_ (2) *bla*_SHV-5-like_ (3) *bla*_SHV-12_ (1) *bla*_VEB-1_ (1)	*mgrB*-0% (2) *mgrB*-53 % (1) *mgrB*-60 % (1) *mgrB*-62 % (3) *mgrB*-87 % (1)	*ybt* 10; ICEKp4 (*n*=2) *ybt* 16; ICEKp12 (*n*=2)	*bla*_KPC-2_ and Tn*4401a-1* (*n*=5)
ST258 (*n*=108)	*bla*_KPC-2_ (99) two copies of *bla*_KPC-2_ (1) *bla*_KPC-3_ (4) *bla*_KPC-23_ (3)	*bla*_SHV-12-like_ (17) *bla*_VEB-1_ (3) *bla*_CTX-M-15_ and *bla*_VEB-1_ (1)	*mgrB*-60 % (61) *mgrB*-62 % (5) *mgrB*-0 % (1) *mgrB*-49 % (1) *mgrB*-6 % (1) *mgrB*-60 % and *pmrB*-76 % (1)	*ybt* 13; ICEKp2 (*n*=92) *ybt* 13; ICEKp2 (truncated) (*n*=5)	*bla*_KPC-2_ and Tn*4401a-1* (*n*=88) *bla*_KPC-2_ and Tn*4401a-*unknown (*n*=11) two copies of *bla*_KPC-2_ and Tn*4401a-*unknown-1 (*n*=1) *bla*_KPC-23_ and Tn*4401a*-unknown (*n*=3) *bla*_KPC-3_ and Tn*4401a-2* (*n*=4)
ST11 (*n*=18)	*bla*_NDM-1_ (17) *bla*_NDM-9_ (1)	*bla*_CTX-M-15_ (15) *bla*_CTX-M-15_ and *bla*_VEB-1_ (1)	*mgrB*-60 % (7) *mgrB*-15 % (2)	*ybt* 15; ICEKp11 (*n*=17) *ybt* 15; ICEKp11 (truncated) (*n*=1)	–

### AMR gene makeup of the CRKP strains

Outside of the carbapenemases, 30 % of the main lineages also carried a variety of extended-spectrum beta-lactamase (ESBL) genes (Table 1, Fig. S2). In 100 CRKP strains (out of 209), we identified truncations of varying lengths in the *mgrB* gene, which has been previously associated with colistin resistance [43]. In MgrB mutations, the frameshift mutation causes a shift in the reading frame of the gene, resulting in a premature stop codon and a truncated, non-functional protein. Of these 100 strains, however, only 53 (53 %) exhibited phenotypic resistance to colistin (Table S3, Fig. S1). Of note, another 20 CRKP strains also displayed resistance to colistin in the absence of *mgrB* mutations or an acquired *mcr* (Table S3). We therefore cannot find a good correlation between *mgrB* truncation and colistin resistance (Fig. S1B). We also did not find any correlation between the degree/extent of *mgrB* truncation and colistin resistance phenotype. On screening for other colistin resistance genes (e.g. *mcr*) or mutations in colistin resistance genes previously reported (e.g. *phoQ*, *crrA*, *crrB*, https://www.ncbi.nlm.nih.gov/pathogens/refgene/#colistin) [47], none of the mutations or genes (e.g. *mcr*) were observed. Genomic prediction of this phenotype is known to be difficult, and further genomic changes such as mutations are still to be identified. A high concordance between phenotypic resistance and genotypic resistance to ciprofloxacin was observed (94 %) as 197 of 199 resistant strains were predicted to harbour genes/gene mutation conferring resistance to ciprofloxacin (Table S3). Interestingly, ten ciprofloxacin-susceptible strains were predicted to harbour resistance genes, lending credence to the fact that several factors (e.g. gene regulatory mechanisms) might affect gene expression. In addition, low concordance between phenotypic and genotypic resistance to trimethoprim (79.9 %), amikacin (78.5 %) and gentamicin (38.2 %) was observed (Table S3), thereby suggesting that a comprehensive understanding of the mechanisms or resistance to these antibiotics are needed for accurate detection of resistance phenotypes. Apart from the yersiniabactin genomic island, we could not identify any markers of hypervirulence in any of the isolates (Table 1).

### Evidence of CRKP transmission within the hospital and description of lineages

#### ST147

We reconstructed the phylogeny of ST147, and found that two main expansions can be seen, one with isolates in the first detection period 2003–2010 carrying *bla*_VIM_, and a later expansion of isolates between 2014 and 2018, where also *bla*_KPC-2_ and *bla*_NDM_ genes were found. With this split was also a change in the predominance of carbapenemases, as the first expansion nearly exclusively carried *bla*_VIM_, whereas the most recent expansion shows a variety of carbapenemases, *bla*_VIM_, *bla*_NDM_ and *bla*_KPC_.

**Fig. 3.**
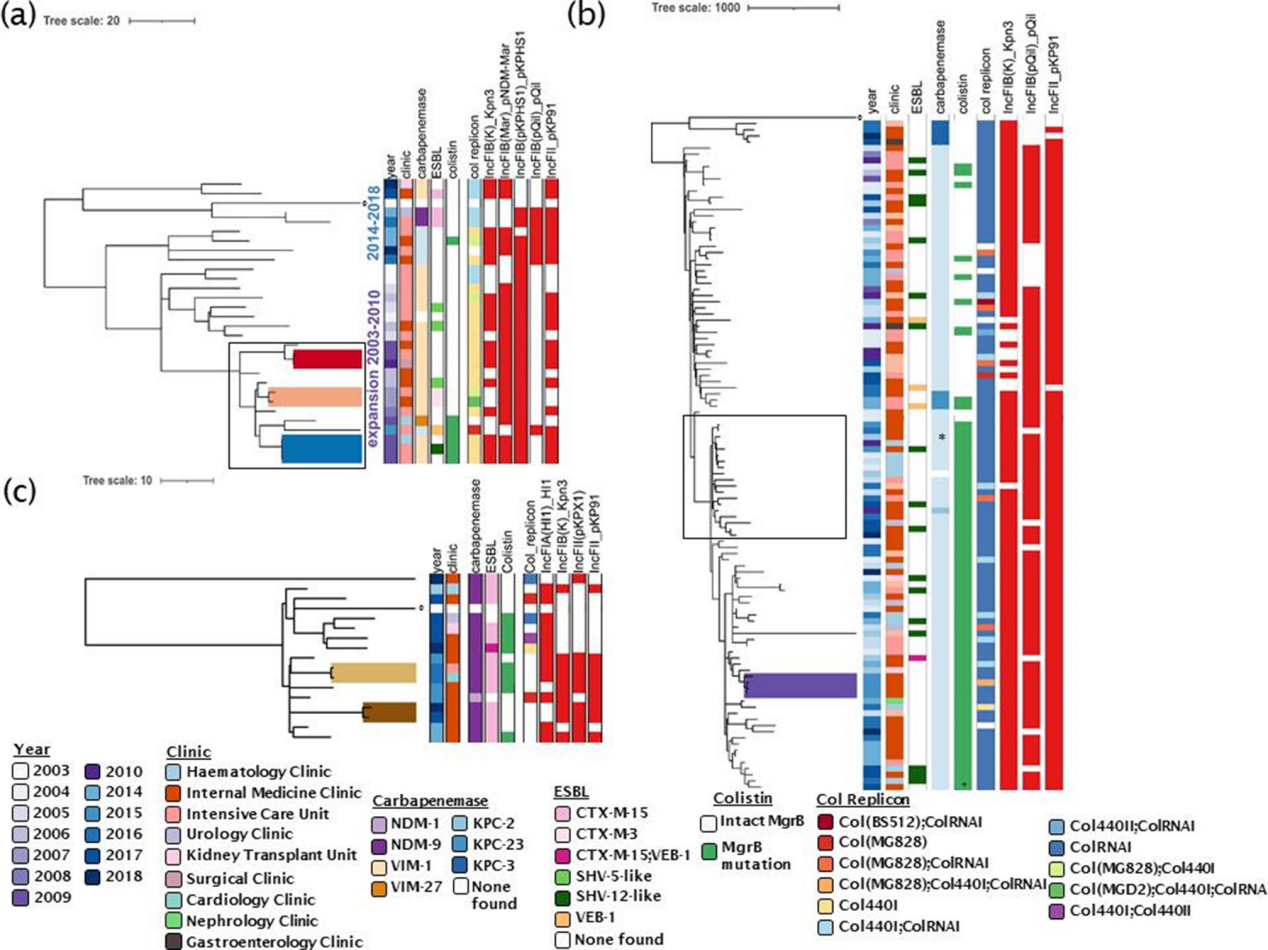
Gubbins-generated phylogenetic trees and metadata showing the genomic characteristics of the major clonal complexes. (**a**) ST147 isolates recovered between 2003 and 2018 (clades depicting the first, second and third putative transmission events are coloured red, khaki and blue, respectively). (**b**) ST258 isolates recovered from 2006 to 2018 (transparent box depicts locally circulating lineage, coloured clade the putative transmission event). (**c**) ST11 isolates recovered from 2013 to 2018. Coloured clades signify putative transmission events. *The ST258 isolate with two copies of the *bla*_KPC_ gene; ^+^the ST258 isolate with *mgrB* mutation and *pmrB* mutation; reference genomes are highlighted with °.

We detected three putative transmission events retrospectively (Fig. 3a, coloured clades), based on the date of isolation, SNP difference, similarity of AMR gene and plasmid gene profile between clones. The first transmission event involved two *bla*_VIM-1_-carrying strains (along with OmpK35 truncation and OmpK36GD genes) with a single SNP difference that were retrieved from an ICU patient and a surgical ward patient about a year apart (ICU: February 2009; Surgical Ward: April 2010, Fig. 3a, red-coloured clade). These two genomes were 11 SNPs different from their nearest neighbour – a strain recovered from a patient admitted to an internal medicine ward (March 2009). The three strains share the same capsular locus (KL64), lipopolysaccharide (O2v1), AMR profiles and plasmid profiles (Fig. 3a, red-coloured clade). The second putative transmission event involved two strains (co-carrying *bla*_VIM-1_, *bla*_CTX-M-3_, *mgrB* truncation, OmpK35 truncation and OmpK36GD, and similar plasmid replicons except col replicons) separated by five SNPs; one strain was recovered from an ICU patient and the other from an internal medicine ward patient, 18 days apart (Fig. 3a, orange-coloured clade). Like the first transmission event, the closest neighbour (Internal Medicine Clinic) differed from the two genomes by 11 SNPs. The third putative transmission event involved three strains (co-carrying *bla*_VIM-1_, *mgrB* truncation, OmpK35 truncation and OmpK36GD) retrieved within 2 months (June and August 2009) from two ICU patients and one patient from the haematology ward, with zero SNPs difference (Fig. 3a, blue-coloured clade). One of the genomes of the isolates of the third cluster recovered from the ICU patients carried an ESBL-encoding gene, *bla*_SHV-12_. As only bloodstream isolates were collected, it is likely that patients with other infection types or only colonization have been missed. Furthermore, these isolates not only have close neighbours (11 SNPs distant) but also form part of a more loosely connected group 20–100 SNPs apart, which may be an indication of a locally circulating clone (transparent box Fig. 3a).

All ST147 isolates carried mutations in the quinolone resistance-determining regions (*gyrA*-83I and *parC*-80I). More than 90 % (*n*=27/29) of ST147 isolates carried genes conferring resistance to drugs within at least six antibiotic classes. ESBL-encoding genes were sporadically present (12/28 isolates, Table 1, Fig. S2A). We could not determine the plasmid carrying the *bla*_VEB_ as it did not share the same contig with any plasmid replicon type. Nevertheless, mobile genetic elements flanking the *bla*_VEB_ gene were similar in one ST147 strain and five other members of the dominant lineages (four ST258 strains, one ST11 strain; Fig. S3). Previously, an association was reported in epidemic clones of *bla*_VIM_ *–bla*_SHV-5-like_-carrying *K. pneumoniae* in Greece [14], but we only detected *bla*_SHV-5-like_ in three of the 20 *bla*_VIM_-carrying ST147 strains (Fig. 3a).

Apart from three *bla*_KPC-2_-carrying isolates which shared the same contig with IncFII replicons (in two of these strains, IncFII was found on multiple contigs), carbapenemase genes and plasmid replicons were observed almost always on different contigs (Fig. S4A). Interestingly, few acquired virulence genes in the form of yersiniabactin were observed, only in four isolates retrieved, one each in 2014, 2016, 2017 and 2018. Within ST147 genomes, the *bla*_VIM-1_ gene was flanked by mobile genetic elements as well as genes conferring resistance to the aminoglycosides, trimethoprim and quaternary ammonium compounds (Fig. S4A). The *bla*_KPC-2_ and *bla*_NDM-1_ genes, on the other hand, were flanked mainly by transposases in the absence of other AMR genes (Figs S5B and S5C). All *bla*_KPC-2_-carrying ST147 strains carried only one variant (Tn*4401a-1*) of the Tn*3*-based transposon (Table 1). NDM-positive strains harbour the IS*Aba125* element as previously described [48]. The *bla*_NDM-1_ gene, unlike *bla*_KPC-2_, was not carried on the same contig with plasmid replicons.

#### ST258

Similarly, we estimated the phylogeny for the ST258 complex. The earliest isolate of this lineage was found in 2006, earlier than previous reports from Greece suggested [49–51] though it was not detected at the time. No ST258 clone was retrieved in 2007, so we compared ST258 strains retrieved in 2006 (*n*=1) and 2008 (*n*=2) and found them to be 33–37 SNPs apart, which would not be indicative of direct transmission or clonal persistence within this hospital, as with reported mutation rates we would only expect four SNPs per year [52]. Rather, this suggests that circulation outside of Laiko and within other hospitals was occurring, and also quite early upon introduction of this lineage into the country. Similarly, we identified another locally circulating lineage that involved a stable clonal expansion over 8 years (2010–2018), including 19 isolates (12/19 were 20 SNPs distance apart) across seven hospital wards (Fig. 3b, transparent box). We detected one potential transmission event in this retrospective study (Fig. 3b, purple clade), which involved four phylogenetically related isolates (2–15 SNPs) recovered from patients admitted to two internal medicine wards 10 months apart (in 2015).

About 84 % of ST258 isolates carried genes conferring resistance to drugs within at least seven antibiotic classes. Like ST147 isolates, all ST258 isolates had SNPs in the quinolone target genes (*gyrA*-83I and *parC*-80I). Again, ESBL carriage was sporadic, with only 21 isolates carrying ESBL-encoding genes (17, *bla*_SHV-12_; 3, *bla*_VEB-1_; 1, *bla*_CTX-M-15_ and *bla*_VEB-1_) (Table 1, Fig. S2B). We were able to identify the plasmid location by co-carriage of *bla*_KPC-2_ and the IncFII replicon on the same contig in 22 (~20 %) ST258 strains and of three other strains carrying *bla*_KPC-3_ and the IncX3 replicon on the same contig (Fig. S4B). Within ST258 genomes, the *bla*_KPC-2_ and *bla*_KPC-3_ genes were mainly flanked by mobile genetic elements (Figs S6A and S6B). Almost all the typical components (*tnpR*, *tnpA*, *istB*) of the transposon Tn*4401a-1* (involved in *bla*_KPC_ gene mobilization [53]) were observed in *bla*_KPC-2_-carrying ST258 isolates (Fig. S6). The *bla*_KPC_ gene is known to be associated with different IncF plasmids [54] and in this collection we found support of this, as different IncFIB and IncFII plasmids can be detected, but none of which is present in all isolates, suggesting movement of the transposon within this population. In the isolates with a KPC outside the ST258/512 lineage, we detected the IncFIB(pQiL) replicon in 14 of 16 isolates, with other IncF1B replicon types in the other two isolates, but we cannot determine whether the carbapenemase is carried on those plasmids. In contrast, the *bla*_KPC-3_-carrying lineage of ST258 is closely associated with carriage of the Tn*3*-based transposon variant Tn*4401a-2* by these strains.

#### ST11

Apart from one outlying isolate, ST11 isolates from Laiko hospital form a homogenous group within 2–100 SNPs difference. Two putative transmission events were suggested retrospectively (Fig. 3c). The first event involved two isolates recovered from two different wards (ICU and Gastroenterology) that shared the same AMR gene profile, and plasmid gene profile, and were within two SNPs. They were also recovered 3 months apart (March and July 2016, Fig. 3c, light brown clade). The second putative transmission event involved two other isolates that shared similar AMR, virulence and plasmid gene profiles, had a 7 SNP distance and were recovered 1 year apart (2017–2018) from two internal medicine wards (Fig. 3c, dark brown clade).

All strains belonging to ST11 carried the yersiniabactin virulence gene (virulence score=1). ESBL carriage was more prevalent in this lineage, as 16 of 18 isolates carried *bla*_CTX-M-15_ (Table 1, Fig. 3c). One isolate additionally also had the *bla*_VEB-1_ gene, but no plasmid replicon was associated with it. The plasmid replicons IncFIA(HI1)_HI, IncFIB(K)_Kpn3, IncFII(pKPX1) and IncFII_pKP91 were the most common plasmid replicons carried by ST11 isolates. Of these, four strains carried all the aforementioned plasmid Inc types. The *bla*_NDM-1_ carbapenemase and plasmid replicons were sometimes carried on the same contig (Fig. S4C), with the main association on the same contig with the plasmid replicon IncFIA(HI1) in seven (39 %) of the ST11 isolates, and the IncFII (pKPX1) plasmid replicon in three of the ST11 isolates (Fig. S4C). Mobile genetic elements, the bleomycin resistance gene and several hypothetical proteins mainly flank the *bla*_NDM_ genes (Fig. S7), akin to the genetic environment observed in *bla*_NDM_-carrying ST147 strains (Fig. S5c). As observed in *bla*_NDM_-carrying ST147 strains, all *bla*_NDM_-carrying ST11 strains (regardless of the *bla*_NDM_ allele) carried the IS*Aba125* element.

## Global context of the dominant CRKP lineages

### ST147 in Laiko hospital mirrors the global diversity of the ST147 lineage

**Fig. 4.**
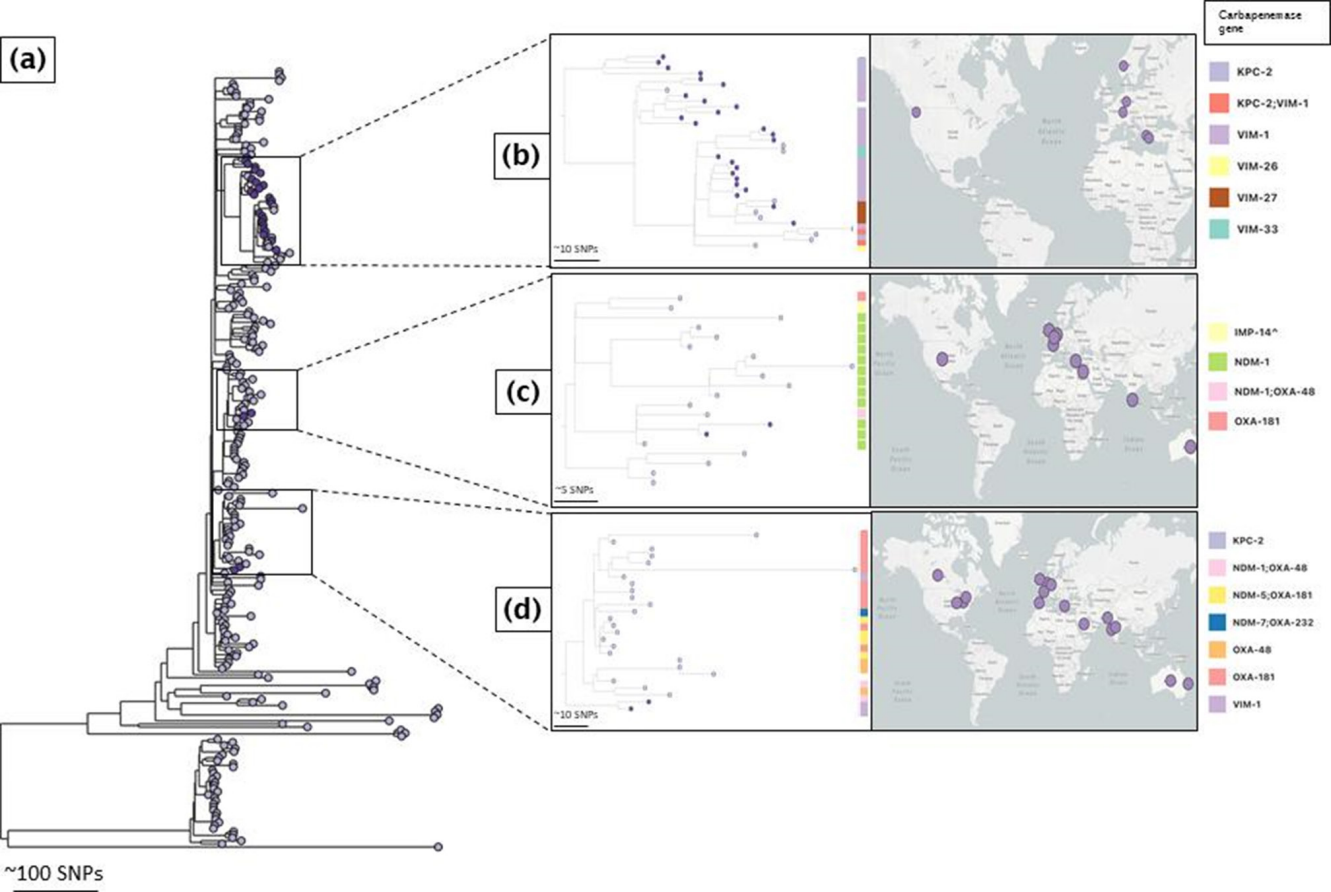
A global phylogenetic tree of *K. pneumoniae* ST147 genomes. (**a**) The three distinct clusters (dark purple tree nodes) formed by ST147 isolates in this study. (**b**) The top cluster comprises genomes from this study (dark purple tree nodes) and genomes from Europe and the USA (light purple tree nodes). (**c**) The middle cluster comprises genomes from this study (dark purple tree nodes) and genomes from regions in North Africa, Europe and North America (light purple tree nodes). (**d**) The bottom cluster comprises genomes from this study (dark purple tree nodes) and genomes from Australia, South Asia, Europe and North America (https://pathogen.watch/collection/pll287yfjynm-daikos-greece-st147-and-global-collection; https://microreact.org/project/sBQBwnNFwemsJS3GkgdUMk-st147-daikos-global-context). Legends show the type of carbapenemase genes carried by isolates in this study and global *K. pneumoniae* ST147 genomes belonging to the same sub-lineage.

When placed within the context of globally disseminated ST147 isolates, the ST147 genomes formed three distinct clusters along the global phylogenetic tree (Fig. 4a). The top cluster was the largest, encompassing 25 ST147 genomes from this study and genomes from Greece, Switzerland, Germany, Norway and the USA (Fig. 4b, Table S4). This cluster itself is an aggregation of three sub-clusters: the top sub-cluster comprises only *bla*_KPC-2_-carrying isolates, the middle sub-cluster comprises *bla*_VIM-1_-carrying genomes, and the bottom sub-cluster comprises *bla*_KPC-2_ and diverse *bla*_VIM_-carrying isolates. The transmission chains detected in the local ST147 isolates are all part of this last cluster. The middle cluster in the overview tree encompassed two *bla*_NDM-1_-carrying genomes from this study and *bla*_NDM-1_-carrying isolates (sometimes co-carried with *bla*_OXA-48_) from Egypt, France, the Netherlands and the UK (Fig. 4c). The bottom cluster comprises two *bla*_VIM-1_-carrying genomes from this study and genomes carrying diverse carbapenemase genes from Australia, Saudi Arabia, India, Spain, France, the Netherlands, USA and Canada (Fig. 4d). Overall, this shows that the local ST147 population reflects the global ST147 population diversity.

### ST258 is dominated by the original first introduction into Europe

**Fig. 5.**
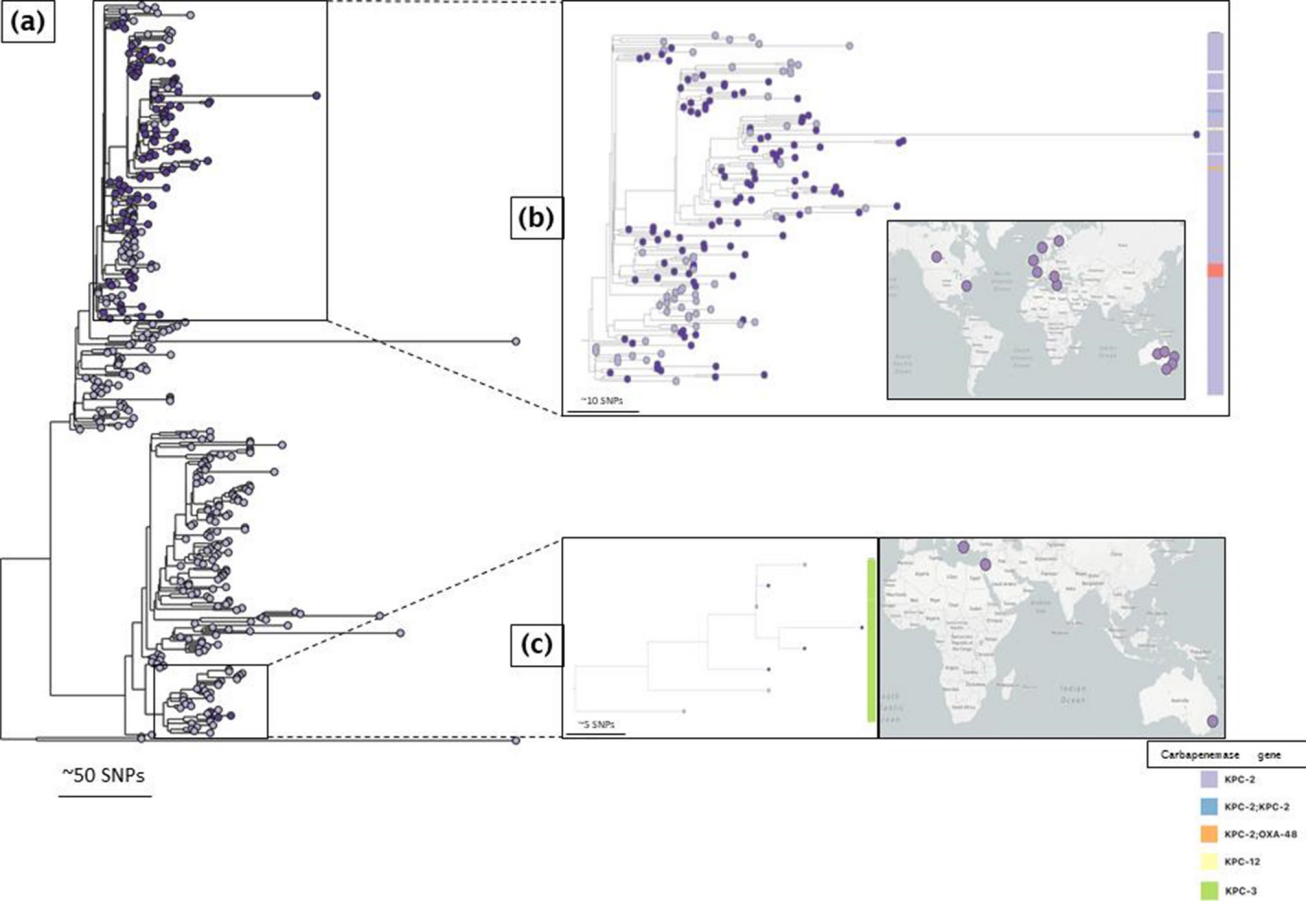
A global phylogenetic tree of *K. pneumoniae* ST258 genomes. (**a**) The two different clusters (dark purple tree nodes) formed by ST258 genomes in this study. (**b**) The top cluster (Cluster 1) comprises *bla*_KPC-2_-carrying genomes from this study (dark purple tree nodes) and *bla*_KPC-2_-carrying genomes from Australia, Europe and North America (light purple tree nodes). (**c**) The second cluster (Cluster 2) comprises *bla*_KPC-3_-carrying genomes from this study (dark purple tree nodes) and *bla*_KPC-3_-carrying genomes from Israel and Australia (https://pathogen.watch/collection/l8paxhiqfa1f-daikos-greece-st258-and-global-collection; https://microreact.org/project/cbq9gSPtNV89yeXf1igjUZ-st258-daikos-global-context). Legends show the type of carbapenemase genes carried by isolates in this study and global *K. pneumoniae* ST258 genomes belonging to the same sub-lineage.

In a global context, the ST258 genomes of the present study separated into two different clusters along the global phylogenetic tree (Fig. 5a). Genomes within the topmost cluster (cluster 1) were phylogenetically related to the first dissemination of *bla*_KPC-2_-carrying isolates out of the USA (Fig. 5b), whereas *bla*_KPC-3_-carrying genomes within the second cluster (cluster 2, 2016–2018) were closest to *bla*_KPC-3_-carrying isolates from Israel (2011, 2013) that represent a subsequent expansion from Israel to other European countries (Fig. 5c, Table S5).

Although only one putative transmission event was observed, ST258 isolates in this hospital were largely diverse and were primarily dominated by *bla*_KPC-2_-carrying clones that expanded from the original introduction of ST258 into Europe. The temporally later lineages of the complex, signified by a change to KPC-3 and change to ST512 [9], are of little importance locally as well as in Greece overall.

The locally circulating clone with 30–60 SNPs distance is also separate from other global genomes, except three isolates from the UK and two from Australia (Fig. 5b, central clade). The position of the UK and Australian isolates within the phylogenetic tree suggests that these clones emerged via two separate introductions into these two countries from Greece.

### ST11 is geographically restricted

**Fig. 6.**
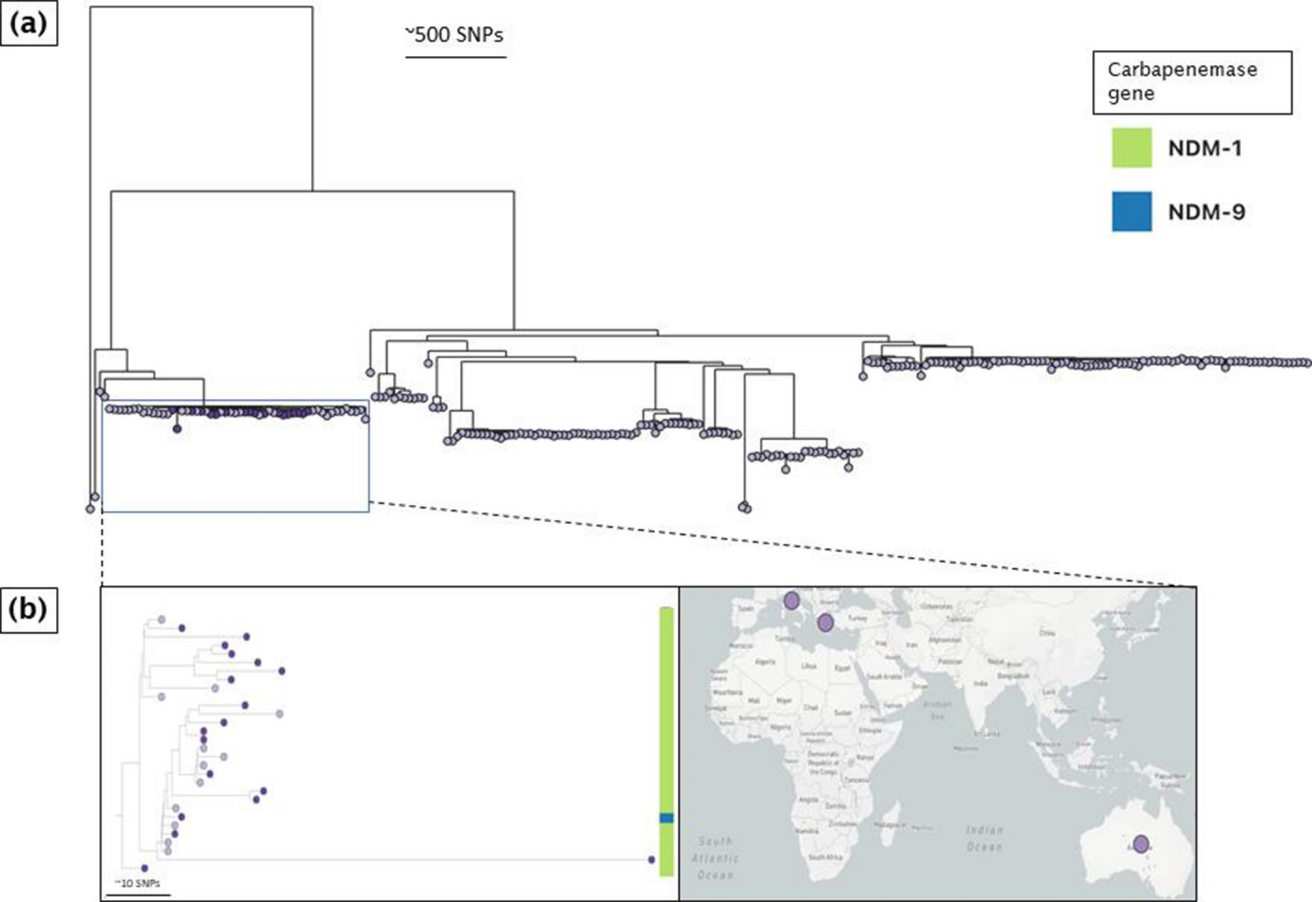
A global phylogenetic tree of *K. pneumoniae* ST11 genomes. (**a**) The localized cluster formed by ST11 genomes in this study (dark purple node), (**b**) along with genomes submitted from Italy and Australia (light purple node) (https://pathogen.watch/collection/ezyujarj6aw5-daikos-greece-st11-and-global-collection; https://microreact.org/project/g7ykirHAVyFxETufRPQJnh-st11-daikos-global-context). Legends show the type of carbapenemase genes carried by isolates in this study and global *K. pneumoniae* ST11 genomes belonging to the same sub-lineage.

In the context of globally ST11 isolates and in contrast to ST147 or ST258, all Laiko genomes clustered together in one lineage (Fig. 6a), thereby suggesting a local expansion of ST11, rather than introduction events from other parts of the world. Global ST11 genomes that clustered within the same lineage as the ST11 CRKP pathogens were retrieved from Italy and Australia indicating limited exchange (Fig. 6b, Table S6). Overall, the ST11 isolates observed in the Greek hospital appeared much later than ST147 and ST258 within the sampling period and represent a geographically and temporally restricted clone.

## Discussion

Herein, we have identified and characterized the major CRKP clones of epidemic importance within a Greek hospital between 2003 and 2018. The majority of isolates belonged to three major clonal lineages, ST258, ST147 and ST11, and the main mechanism of carbapenem resistance was acquired carbapenemase genes (*bla*_KPC_, *bla*_VIM_ and *bla*_NDM_, respectively). *bla*_VIM_-carrying ST147 emerged at the start of the sampling period (2003), followed by the dominant *bla*_KPC-2_-carrying ST258 from 2006 onward, and *bla*_NDM-1_-carrying ST11 in 2013 and thereafter. Since the first detected isolates of each carbapenemase were included in the study, we can be certain about their date of emergence within Laiko hospital. These time periods mirror the date of emergence of these clones in Greece [49–51], which spread rapidly within tertiary referral centres after their first appearance in this region. In particular, the ST258 lineage carrying *bla*_KPC-2_ was detected at a slightly earlier date in our isolates, which went unnoticed at the time. The genetic distance between two isolates 2 years later also indicates that this clone was potentially circulating extensively by this time already.

Their swift dissemination might have been aided by circulation of patients among hospitals due to rotation of hospitals being ‘on call’ in large cities. Given that 5–6 % of new admissions in our hospital are colonized with CRKP [55], it is possible that new CRKP clones are introduced into a hospital by influx of unrecognized colonized or infected patients and sustained by further intra-hospital transmission due to inadequate infection control practices. It is also possible that carbapenemase genes are also disseminated across lineages via self-transmissible plasmids or other mobile elements [14, 56, 57]. Indeed, we observed *bla*_KPC-2_ and *bla*_NDM-1_ across all major lineages carried on the same contig with diverse plasmid replicons, and *bla*_KPC-3_ on IncX3 replicons, supporting the notion that carbapenemase might be transferred within the same and among different CRKP lineages on different plasmids [44, 45]. Furthermore, similarly the genetic background of *bla*_NDM_ in ST11 and ST147 lineages suggests that this gene may be shared within and between *K. pneumoniae* lineages.

With ST258 still being the most prevalent clone in several locations worldwide as well as in our collection, KPC production remains the dominant mode of carbapenem resistance in Europe, and North and South America [6, 9]. Since the first emergence of *bla*_KPC_-carrying ST258 in the USA, it spread into Europe presumably via Israel and Greece [58]. Unlike Italy and Israel, where the predominant carbapenemase is KPC-3, 93 % of our ST258 strains carried *bla*_KPC-2_ and only a few ST258 strains harboured *bla*_KPC-3_ or other *bla*_KPC_ variant [59]. Unlike ST258 and ST11 clonal groups that carried exclusively KPC and NDM carbapenemase genes respectively, the ST147 clone harboured varying carbapenemase genes: VIM, KPC or NDM. It is worth mentioning that the ST147 clonal group is emerging globally as an important vehicle for dissemination of a variety of AMR genes [60, 61].

A substantial proportion of the three major CRKP clones harboured genes conferring resistance to at least six different antibiotic classes. Apart from carbapenem resistance, colisitin resistance is worrisome, as it is one of the ‘last line agents’ for the treatment of CRKP infections. A steady increase in the frequencies of resistance to this drug is already evident after its extensive use [62]. In a previous report from the same hospital, using time series analysis over a 15 year period, a strong association between colistin use and resistance has been observed [63]. Mutations in the *mgr* gene among CRKP with frequencies ranging from 27 to 73.9 % have been observed in previous studies from Greece [51, 52]. We observed mutations in *mgrB* in 47.8 % of CRKP isolates, but a correlation with actual phenotype resistance was not given. Also, approximately one-third of the isolates carried ESBL-encoding genes, including the Vietnamese ESBL *bla*_VEB-1_. None of the isolates, however, harboured the recently described variant, *bla*_VEB-25_, that confers resistance to ceftazidime-avibactam – a novel β-lactam/β-lactamase inhibitor combination [64, 65]. Resistance to ceftazidime-avibactam was observed in one KPC-23 variant with mutations in OmpK35 that was isolated 4 years prior to introduction of this agent in Greece.

Although CRKP bloodstream infections have been occurring during the study period with incidence ranging from 0.04 to 0.35 per 1000 patient-days, no large-scale outbreak was detected within the studied hospital. Given that the analysis included only a proportion of blood isolates and not all clinical isolates and no isolates derived from active screening, there are too many missing points in a putative transmission chain. Therefore, we are unable to state with absolute confidence that no outbreaks occurred in the hospital. Nevertheless, we identified six probable transmission events based on WGS and SNP distances, which indicates that, at least occasionally, transmission from patient to patient was occurring in the hospital.

Our findings should not be interpreted without considering limitations. We examined a proportion (*n*=209) of CRKP and not all consecutive 392 blood isolates. Also, our study did not include other clinical isolates and isolates from colonization to further characterize the evolution of the CRKP epidemic in our hospital. However, we included the first detected isolates of each carbapenemase-producer to capture their introductions into Laiko hospital. Further, the isolates included in the analysis were randomly selected throughout the study period and represent a variety of infections, as 73 % of bacteraemias were secondary to urinary tract infections, hospital- and ventilator-associated pneumonia, and infections from other sources. We also acknowledge the retrospective character of the study and the inherent shortcomings that exist in these types of studies. Finally, for analysis of plasmid-mediated resistance and transposons, long-read sequencing would be better suited to provide a higher resolution.

In the present study, we highlight the importance of a longitudinal survey in investigating the lineages of CRKP and the mechanisms driving AMR within a tertiary referral centre. This could now serve as a springboard in developing strategies to contain the spread of CRKP at the local and national level. We recommend that tertiary referral centres in endemic geographical areas conduct regular surveillance studies on circulating lineages to identify hospital-specific CRKP lineages, detect the emergence of ‘high-risk’ clones promptly, implement hospital-specific infection control measures, and contextualize outbreak strains when present.

## Supplementary Data

Supplementary material 1

Supplementary material 2
