# Supplementary material for: Three Klebsiella pneumoniae lineages causing bloodstream infections variably dominated within a Greek hospital over a 15 year period
Source: Microb Genom. 2023 Aug 29;9(8):mgen001082. doi: 10.1099/mgen.0.001082 (PMC10483420; doi:10.1099/mgen.0.001082)

Supplementary Figure 1

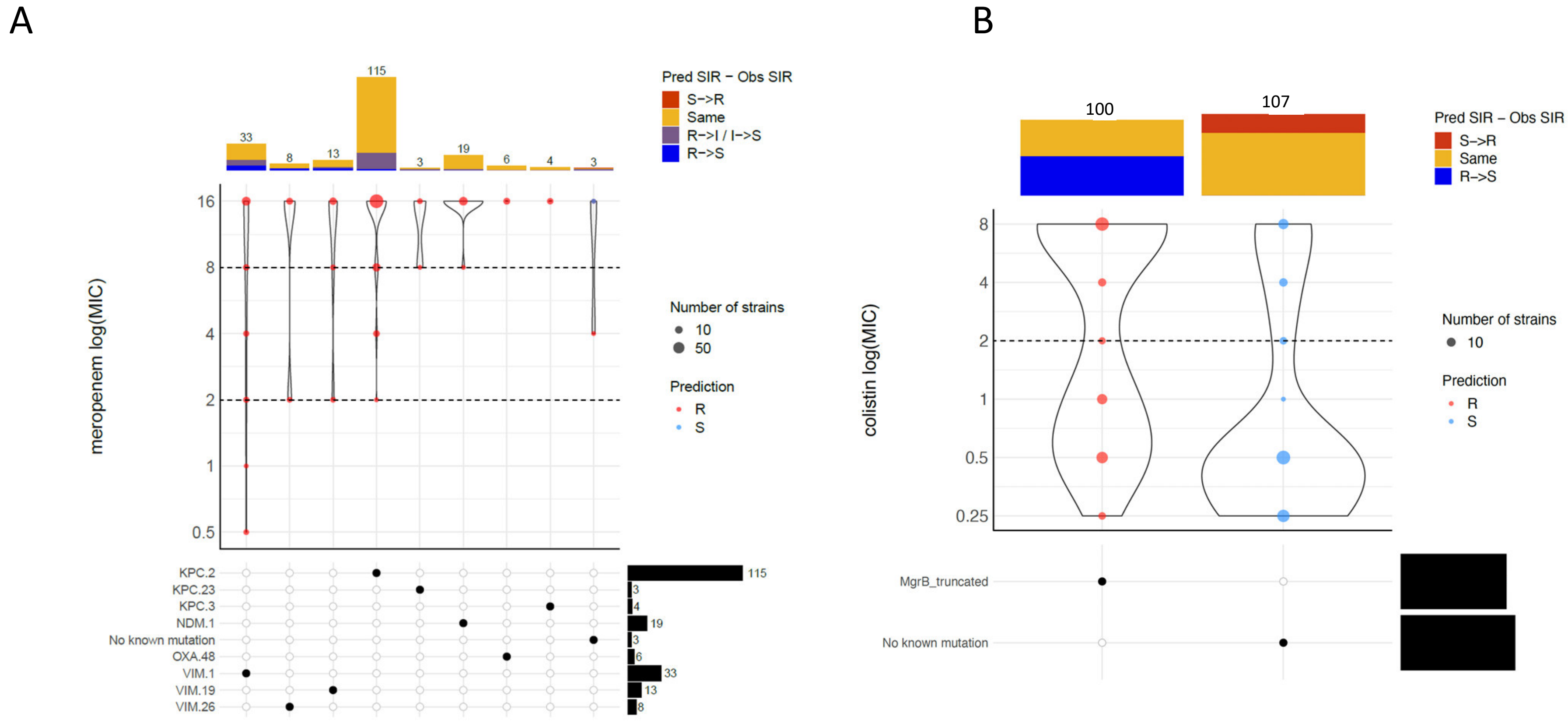

Supplementary Figure 2

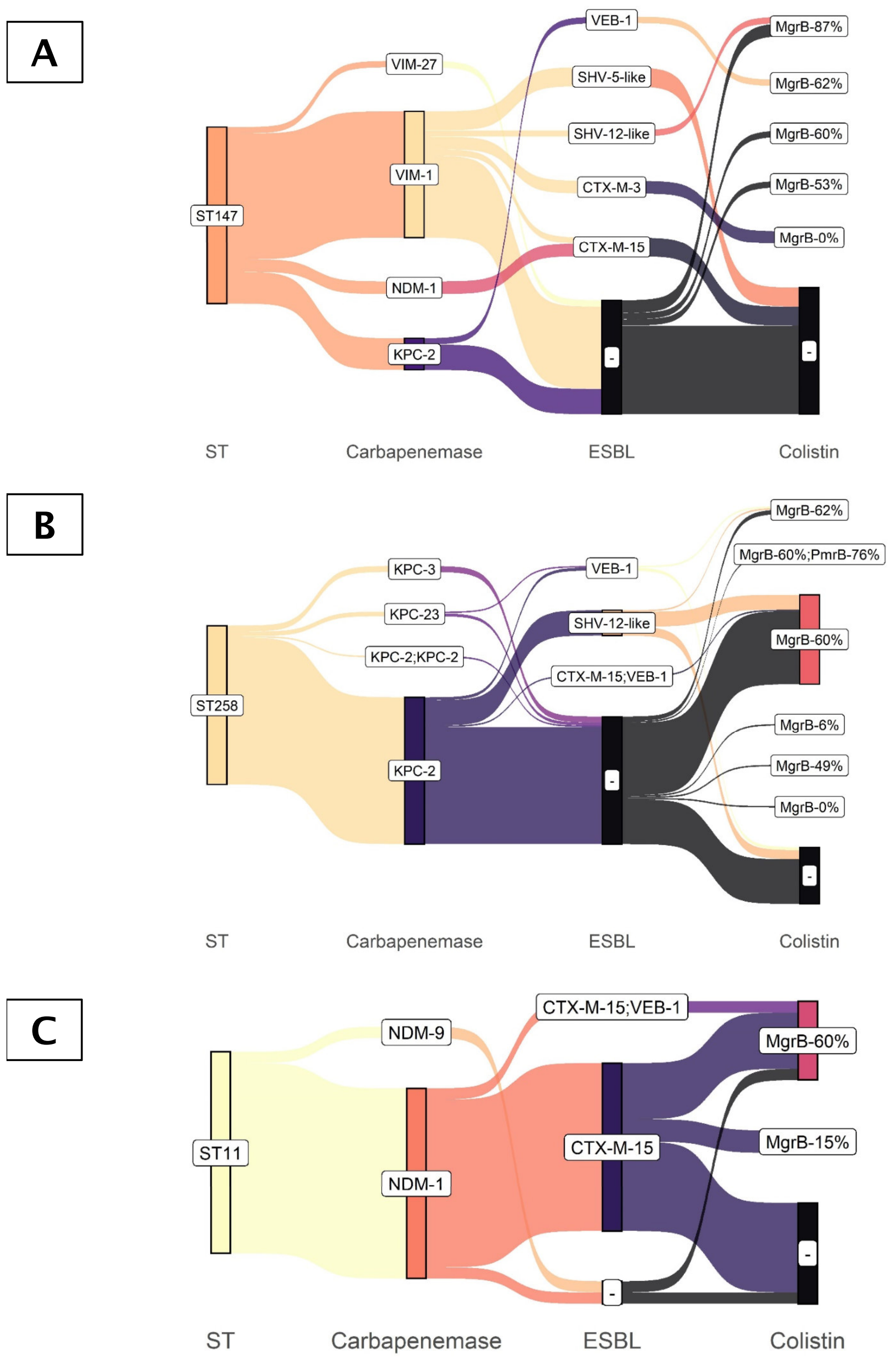

Supplementary Figure 3

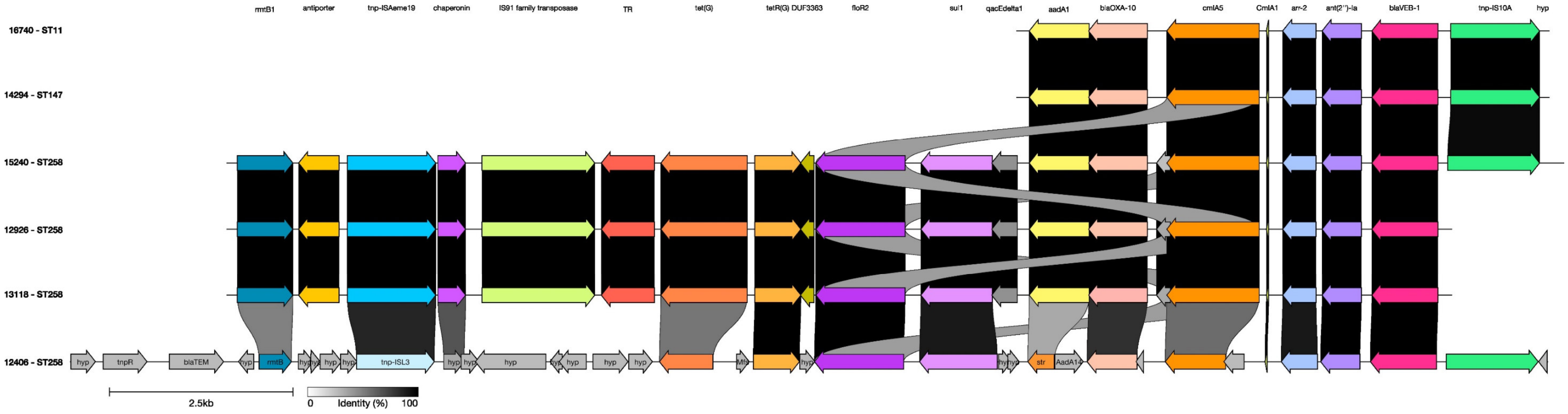

Supplementary Figure 4

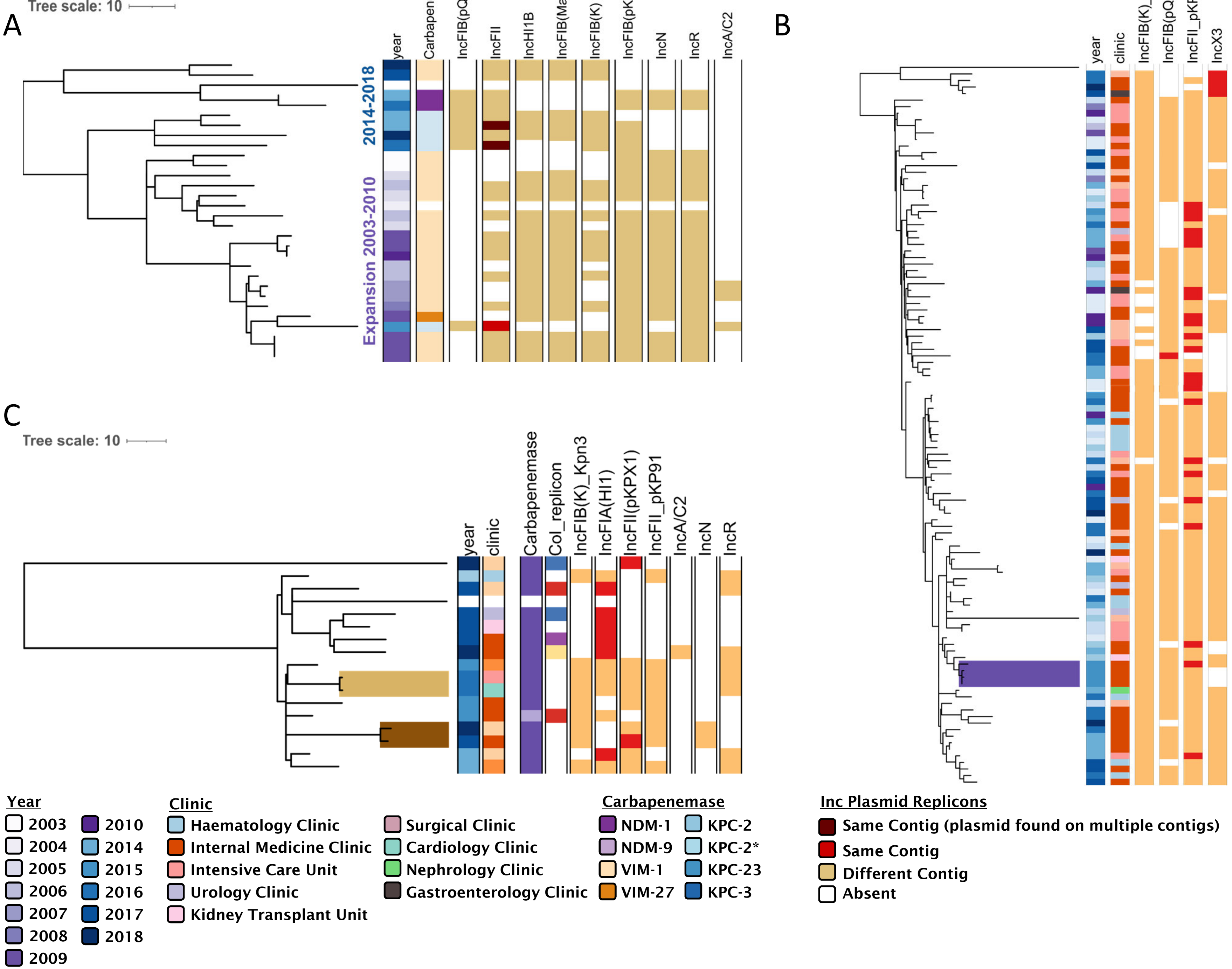

Supplementary Figure 5

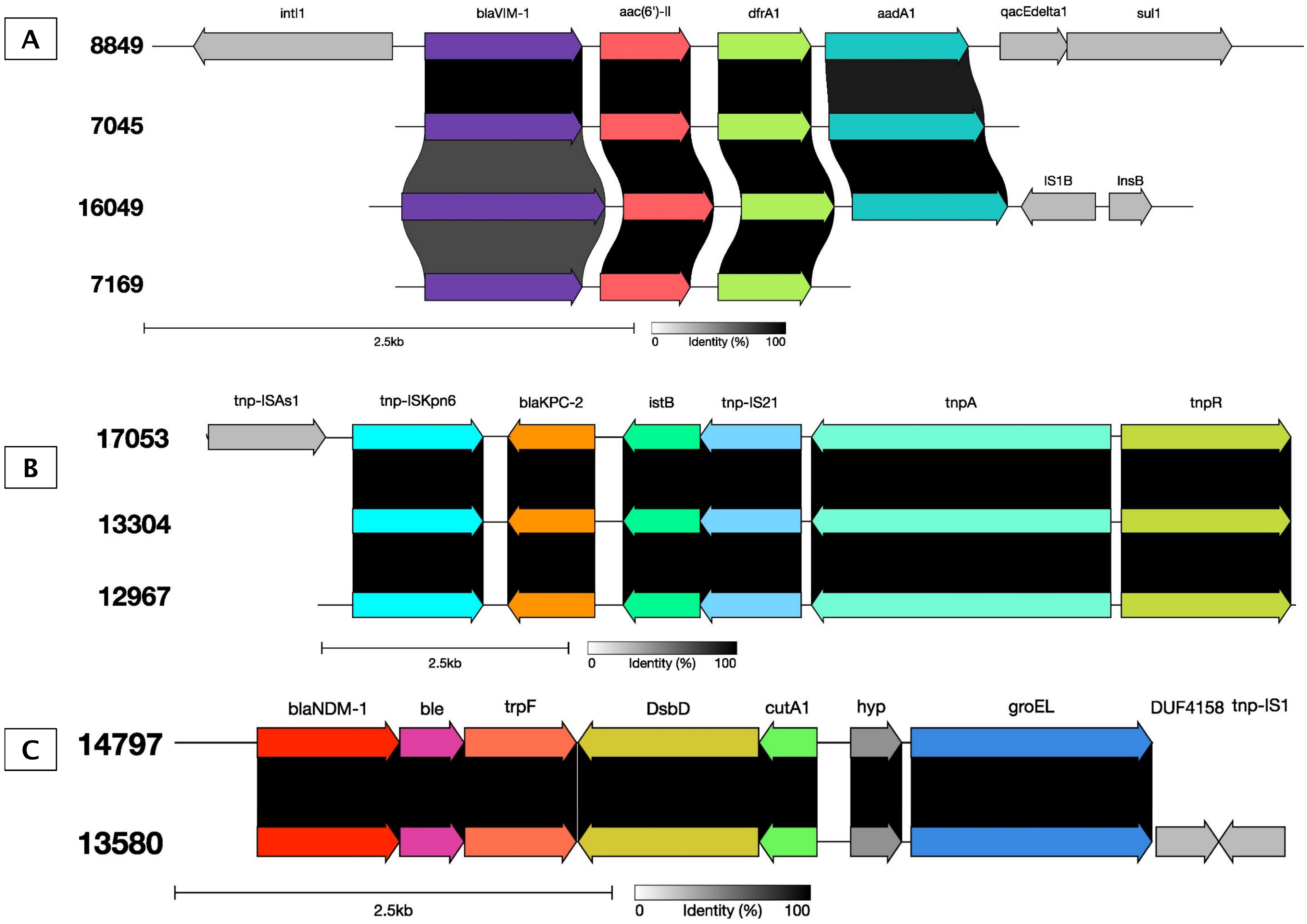

Supplementary Figure 6

A

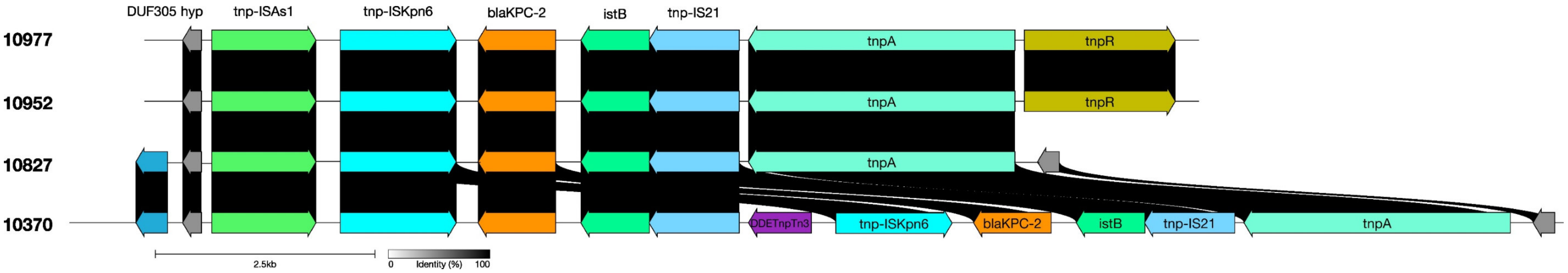

B

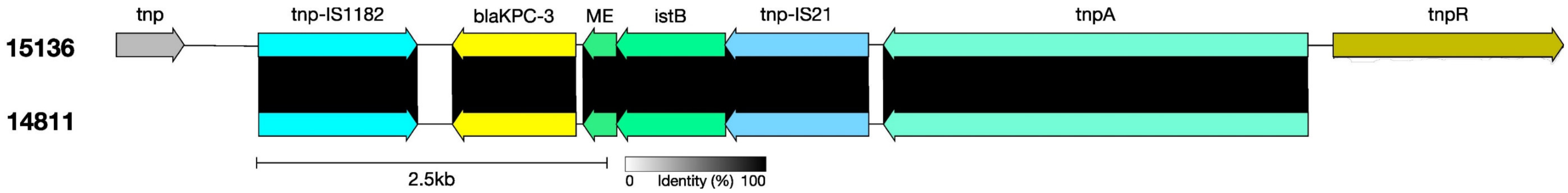

Supplementary Figure 7

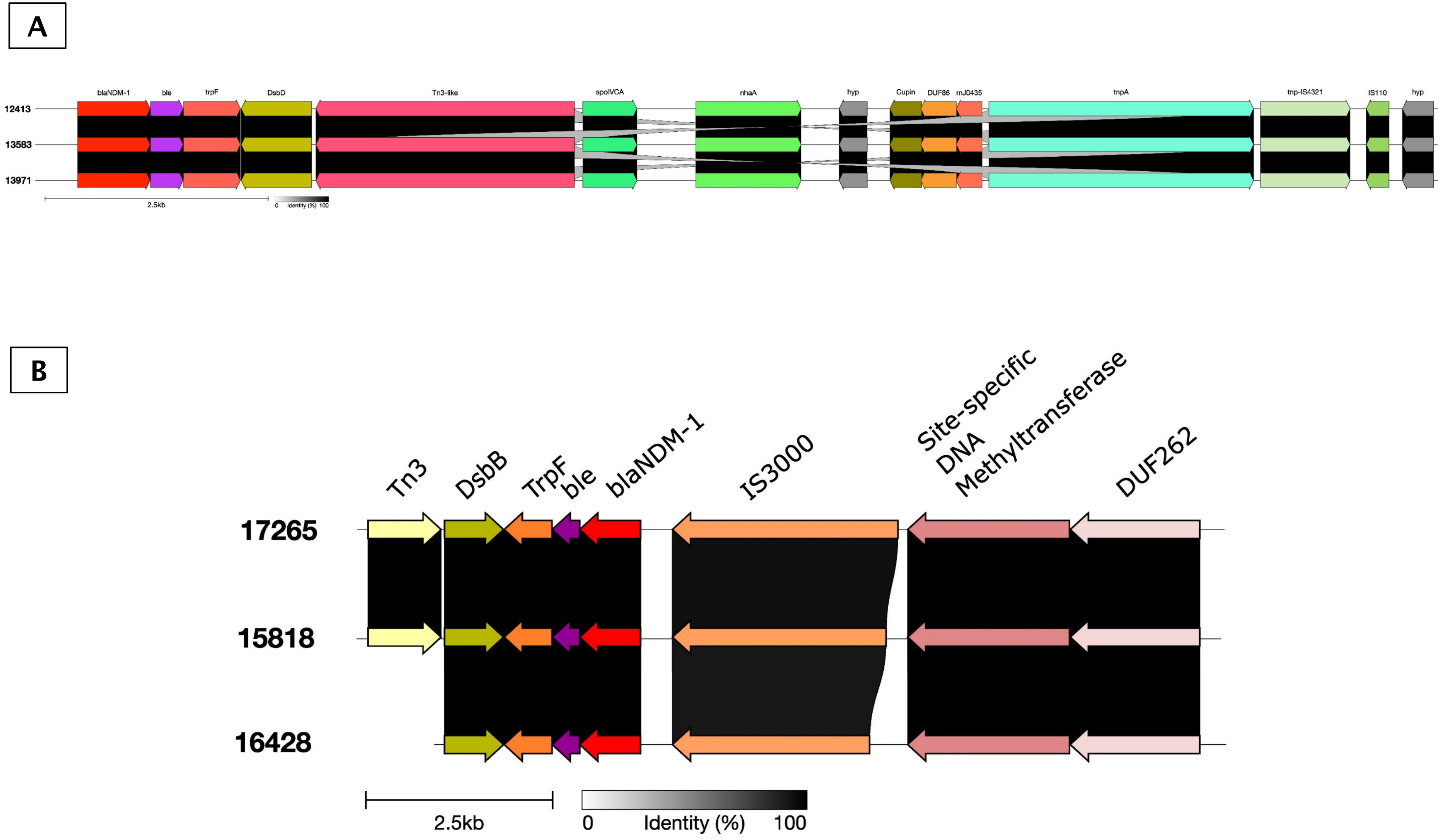

Supplement: Supplementary material 1 [file mgen-9-1082-s001.pdf]
